# C5aR inhibition in the early inflammatory phase does not affect bone regeneration in a model of uneventful fracture healing

**DOI:** 10.1186/s40001-016-0236-7

**Published:** 2016-10-26

**Authors:** Christian Ehrnthaller, Markus Huber-Lang, Anna Kovtun, Anna Elise Rapp, Julia Kemmler, Florian Gebhard, Anita Ignatius

**Affiliations:** 1 Department of Traumatology, Hand-, Plastic-, and Reconstructive Surgery, Center of Surgery, University of Ulm, Albert-Einstein Allee 23, 89081 Ulm, Germany; 2 Institute of Orthopedic Research and Biomechanics, Center of Musculoskeletal Research, University of Ulm, Ulm, Germany

**Keywords:** Fracture healing, Complement, Antagonist, C5aR, C5aRA

## Abstract

**Background:**

Recent studies were able to demonstrate involvement of the complement cascade in bone biology. Further studies analyzed the role of complement in traumatic injuries and demonstrated negative effects after excessive systemic activation of the inflammatory response with early abrogation of complement activation after application of a C5aR-antagonist exerting beneficial effects upon bone regeneration. In contrast, own fracture healing experiments with complement-deficient animals implied a crucial role of the complement cascade for sufficient fracture healing.

**Methods:**

To analyze the effect of a short abrogation of the complement system in the local process of fracture healing, a fracture healing experiment with wild-type mice (C57BL6), femoral osteotomy, consecutive external fixation for 21 days and blockade of the early complement activation (C5aRA) directly after trauma and after 12 h was performed. Control animals received a peptide without any biological effects. After 1–3 days, the inflammatory response was monitored with IL-6 immunostaining, serum analyses of C5a and after 3 days with histological evaluation of PMN. Fracture healing was examined with biomechanical, radiological and histological methods after 21 days.

**Results:**

While a decrease of the early inflammatory response was seen on day 1 of the C5aRA-treated group regarding immunostaining for IL-6 and after 3 days in the histological evaluation of PMN, no significant differences were demonstrated between both experimental groups after 21 days in the biomechanical, radiological and histological evaluation.

**Conclusions:**

The present results demonstrate that the short-term inhibition of complement activation immediately after fracture does not significantly affect bone regeneration in an experimental model of regular fracture healing. Whereas other studies demonstrated that the early posttraumatic blockade of the C5aR improves fracture healing in a scenario of combined trauma, the present findings implicate that the same treatment has no effect in uneventful bone healing.

## Background

The complement cascade represents an essential part of the innate immunity consisting of almost 40 different proteases, which are activated by three established activation pathways (classic, alternative and lectin) in a chain reaction ultimately leading to formation of the terminal attack complex protecting the host from infection [1]. Besides other functions like opsonization of pathogens, the complement system is known to trigger both local and systemic inflammation by activation of the potent anaphylatoxins complement factor 3a (C3a) and C5a [2]. Functions like phagocyte migration, smooth muscle cell relaxation, degranulation of mast cells and basophil granulocytes are mediated after interaction of C3a and C5a with their corresponding receptors C3aR and C5aR [1]. Additionally, Huber-Lang et al. were able to demonstrate cross-activation of the complement system by other serine protease systems (e.g. coagulation system) [3] and also a cellular activation pathway by macrophages has been discovered [4]. Both extrinsic activation pathways have been regarded as an important mechanism in traumatic injuries [3].

Various clinical and experimental studies provided evidence for an involvement of complement in bone biology [5–7]. Besides demonstration of complement activation in bone tissue both locally and systemically after traumatic injuries, the complement system is known to be involved in the pathophysiology of several other bone-affecting diseases like systemic lupus erythematosus (SLE) and rheumatoid arthritis (RA) [8, 9].

Several studies were also able to identify an involvement of the complement cascade in the process of fracture healing [5, 10, 11].

Our group evaluated the role of complement in fracture healing in complement factor 3 (C3)- and C5-deficient mice. Whereas after 21 days healing was successful in the absence of C3, in C5-deficient mice fracture repair was significantly impaired. Therefore, activation of the terminal complement cascade was suggested to be crucial for successful fracture healing [5]. With the most prominent results seen in the early healing phase, the question if complement is crucial for fracture healing throughout all healing phases remained to be elucidated. This problem especially aroused as other studies were able to demonstrate a negative role of complement in the early posttraumatic inflammation phase with impaired fracture healing in a combined severe animal trauma model of blunt chest trauma and femoral osteotomy [10]. In this study, additional intravenous application of a C5aR antagonist (C5aRA) completely abolished the negative effects [11], demonstrating that the complement cascades deleterious systemic effects in a severe multiple trauma model.

Therefore, the question remains, if abrogation of the very early complement activation after a single femur osteotomy with undisturbed fracture healing exerts beneficial or maybe even deleterious effects due to the fact that a balanced inflammation is very important for elimination of alarmins to reduce tissue damage [2].

To further elucidate the involvement of complement in fracture healing in respect to timing and the different fracture healing phases, a fracture healing experiment with systemic administration of a C5aRA immediately after fracture induction was performed.

## Methods

### Mouse models

All experiments followed the international regulations for the care and use of laboratory animals after the approval of the national ethical committee (Germany, Regierungspräsidium Tübingen, No. 965). Surgery was performed under general anesthesia, and all efforts were made to minimize suffering. All mice received analgesic in the drinking water from 2 days preoperatively to 3 days postoperatively (25 mg/l, Tramal®, Gruenenthal, Aachen, Germany).

C57BL/6J mice were chosen for both experimental groups. Mice were purchased from Jackson Laboratories (Bar Harbor, Maine, USA).

### Fracture healing experiments

**Fig. 1:**
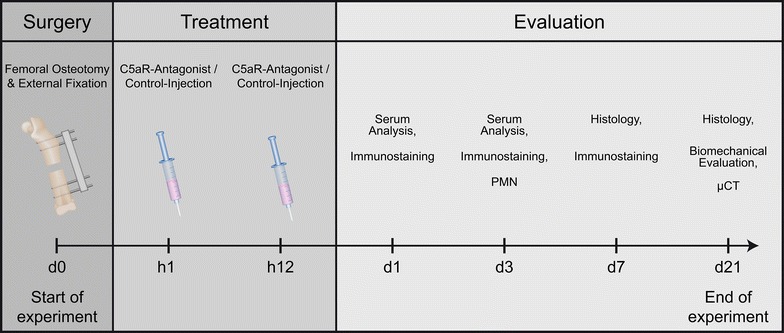
Diagram of the experimental setup with treatment and evaluation time points and the corresponding evaluation methods (µCT = micro-computed tomography; C5aRA = complement factor 5 receptor antagonist)

Fifty male mice, aged 12 weeks, were divided into two experimental groups: femoral osteotomy with administration of a C5aR-antagonist (Fx and C5aRA) (*n* = 24) and femoral osteotomy with application of a control peptide (Fx) (*n* = 26). The surgical procedure was described in detail previously [12]. Briefly, a standardized osteotomy gap of 0.4 mm was created at the midshaft of the right femur and stabilized with an external fixator using 4 mini-Schanz screws (Research Implant System, RIS, Davos, Switzerland). After a healing period of 7 and 21 days, animals were killed. Additional animals were killed for serum and histological analysis on days 1 and 3 (Fig. 1).

### C5aR-antagonist

Immediately after the surgical procedure and femur osteotomy, one group received a C5aR-antagonist (Ac-F [OPdChaWR]; PMX-53) at a dosage of 1 mg/kg intravenously [13, 14]. The injection was repeated 12 h after injury to prevent the C5a-dependent systemic inflammation, which was detectable during the first 12–24 h after the blunt chest trauma in rats [10, 15]. Control animals received a peptide (Ac-F [OPdChaAdR]) with two changed amino acids, which does not have antagonistic activity, and thus, does not develop any biological effect at the same concentration and at the same time points [16].

### Biomechanical testing

All femora explanted on day 21 were subjected to non-destructive three-point bending tests as described previously [12]. Briefly, the proximal femora were embedded in aluminum cylinders using SelfCem (Heraeus Kulzer, Hanau, Germany). Then, the embedded femora were inserted into a material-testing machine (Mod. Z010, Zwick GmbH & Co., Ulm, Germany). The bending load was applied to the top of the callus and was recorded continuously versus sample deflection.

### Micro-computed tomography (μCT)

Femora were scanned using a µCT device (Skyscan 1172, Skyscan) at a resolution of 8 µm and with the settings 50 kV and 200 µA. The region of interest (ROI) was defined as the periosteal callus together with the fracture gap. The callus was segmented and unnecessary callus regions were discarded using a CT-analysis software (Data viewer, Skyscan). To distinguish between mineralized and non-mineralized tissue, a threshold was defined with a grey value corresponding to 25% of X-ray attenuation of the cortical bone of each specimen [17]. To determine the mineral density, bone phantoms with 250–750 mg/kg hydroxyapatite were used for calibration with the same threshold as described above. Common American Society for Bone and Mineral Research (ASBMR) standard parameters including bone volume (BV), tissue volume (TV) and BV/TV (bone volume/tissue volume) were evaluated (CTAnalyser, Skyscan).

### Histological analysis

After fixation in 4% formaldehyde and dehydration with ethanol, the bones were embedded in methyl methacrylate (Merck KGaA, Darmstadt, Germany). Histological slices were harvested from longitudinal sections through the center of the bone and surface-stained with Paragon (toluidine blue and fuchsin; both Waldeck GmbH & Co KG, Münster, Germany). The slices were examined under a light microscope (Leica DMI6000B, Leica, Heerbrugg, Switzerland) at fivefold magnification. The amount of bone, cartilage and fibrous tissue was assessed by circumscribing the corresponding areas using image analysis software (Leica MMAF 1.4.0 Imaging System, powered by MetaMorph1).

### Interleukin 6 (IL-6) immunostaining

After fixation in 4% buffered formalin for 48 h, osteotomized femurs were decalcified using 20% EDTA (ethylene diamine tetraacetic acid) (pH 7.2–7.4) and embedded in paraffin. Immunohistological staining for IL-6 (IL-6 polyclonal rabbit anti-mouse antibody, #bs-0379R, Bioss Inc, Woburn, MA, USA and goat anti-rabbit immunoglobulin G (IgG) secondary antibody, #B2770, Invitrogen Life Technologies GmbH, Darmstadt, Germany) was performed 1, 3 and 7 days after fracture. Respective non-specific IgG subtypes were used as controls. All sections were counterstained with hematoxylin (Waldeck, Münster, Germany) and analyzed under 200- or 400-fold magnification by light microscopy (Leica DMI6000B, Leica, Heerbrugg, Switzerland).

### Immunohistological evaluation of polymorphonuclear leukocytes (PMN)

Polymorphonuclear leukocytes were stained in paraffin-embedded tissue after 3 days with the region of interest defined as the periosteal callus between the inner pins of the fixator. The immunohistological staining and evaluation have been performed as previously described [18].

### Serum C5a measurement

Serum C5a was analyzed after 1–3 days using a commercially available ELISA (enzyme-linked immunosorbent assay) kit (DY2150) in accordance with the manufacturer’s protocol (R&D Systems, Inc., Minneapolis, USA).

### Statistical analysis

Data were expressed as mean ± two times standard error (SEM). Statistical analysis was performed using the unpaired *t* test after testing of normal distribution (IBM SPSS Statistics 19.0, SPSS Inc., IBM, Armonk, New York, USA). Results with *p* ≤ 0.05 were considered significant.

## Results

### Micro-computed tomography (μCT)

Radiological evaluation of the fracture callus 21 days after fracture showed only slight differences between the treatment groups without statistical significance.

**Fig. 2:**
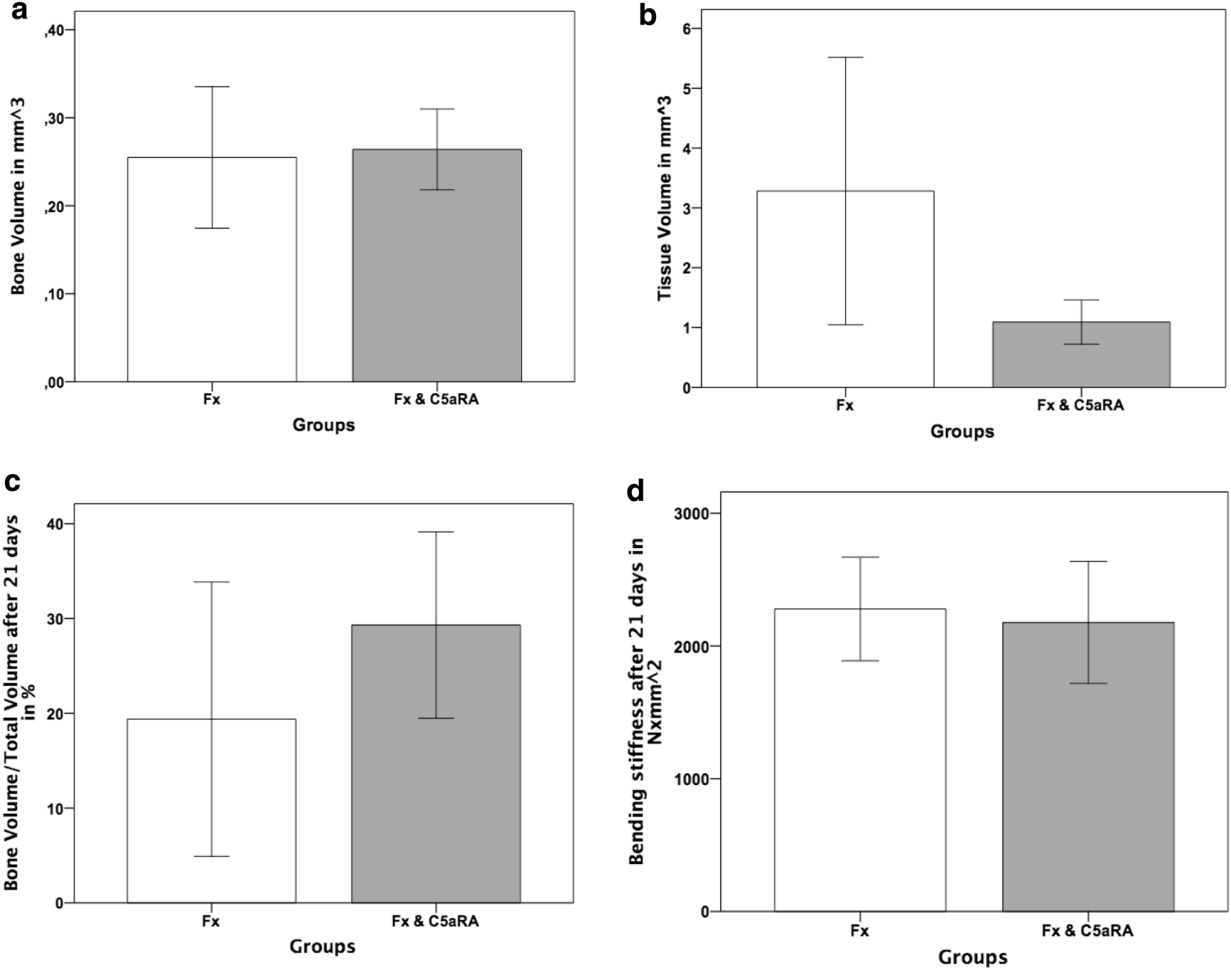
µCT analyses of the fracture callus at 21 days after the osteotomy for both experimental groups (Fx = fracture; Fx & C5aRA = fracture with administration of C5aRA). **a** Bone volume in mm^3^. **b** Tissue volume in mm^3^. **c** Bone volume/total volume in %. **d** Bending stiffness in N × mm^2^. Results are displayed as mean ± 2 × SEM

Whereas the amount of BV was equal between both experimental groups (Fig. 2a; mean bone volume for the fracture group 0.25–0.26 mm^3^ for Fx & C5aRA), the amount of tissue volume (TV) differed 66% with a mean value of 3.3 mm^3^ in the Fx group compared to 1.1 mm^3^ in the Fx & C5aRA group (*p* = 0.99) (Fig. 2b). Consecutively, the value for BV/TV (bone volume/tissue volume) showed slightly higher values (9.9%) for the group of Fx & C5aRA (29.3%) compared to the group of fracture alone (19.4%) (Fig. 2c).

### Biomechanical testing

Evaluation of biomechanical stiffness showed similar values for both experimental groups (Fig. 2d) (Mean value 2279 N × mm^2^ in the fracture group and 2177 N × mm^2^ in the group of C5aRA treatment).

### Histological analysis

**Fig. 3:**
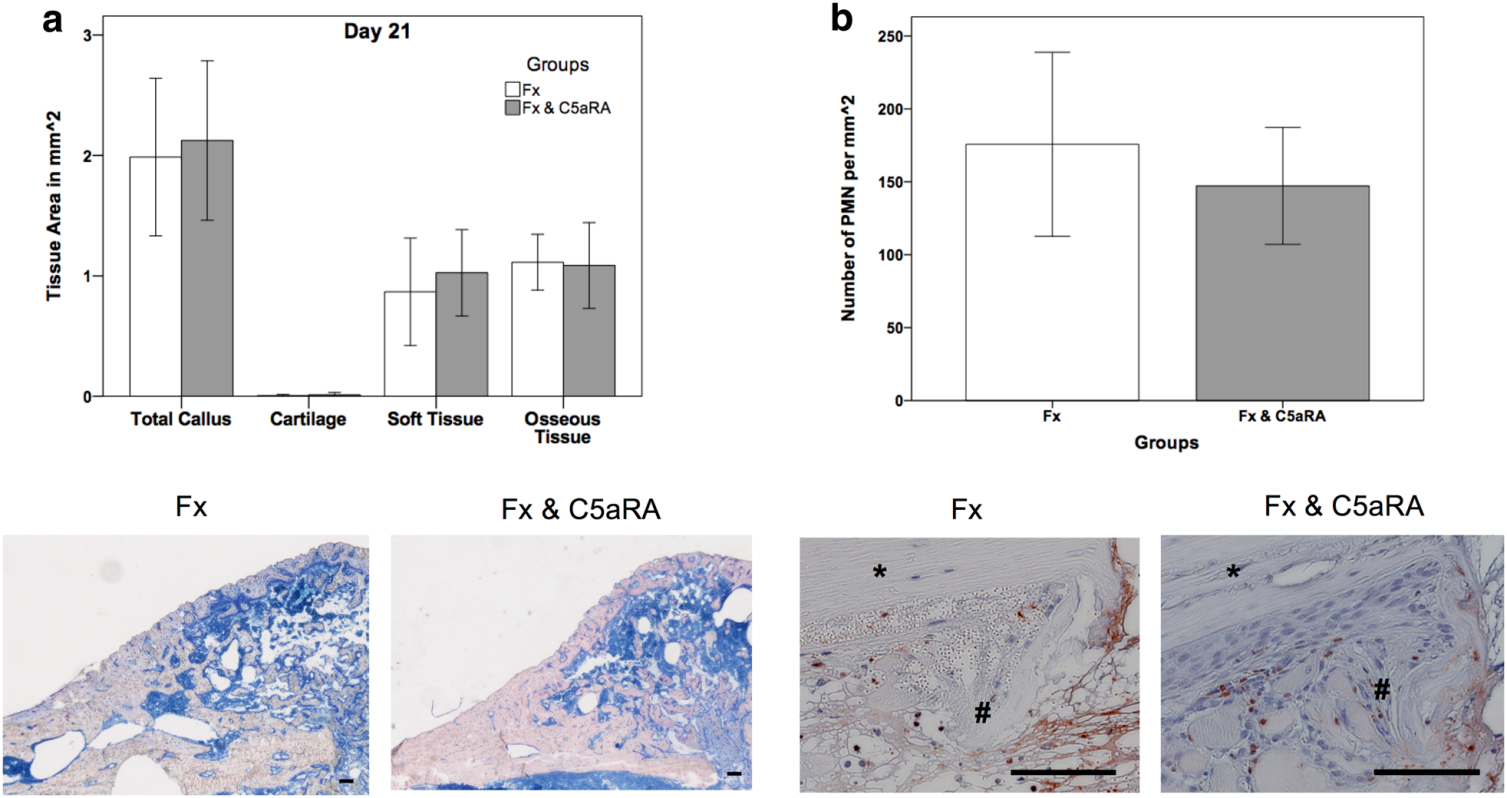
**a** Histological evaluation of the fracture callus for both experimental groups after 21 days with representative histological images of the callus (Fx = fracture; Fx & C5aRA = fracture with administration of C5aRA). **b** Results of histological evaluation of PMN after 3 days with representative histological images of the callus region in detail. Results are displayed as mean ± 2 × SEM; size of the *scale bar* = 100 µm; * = cortex, *#* = periosteal callus

Histological evaluation showed no significant alterations for the different amount of tissue in the fracture callus on both evaluation time points after 7–21 days (Fig. 3a; data from day 7 not shown).

Further histological analysis of PMN after 3 days was not able to depict any significant difference and showed a slightly higher amount of PMN in the control group (16%; *p* = 0.452) than in the group for C5aRA administration (Fig. 3b).

**Fig. 4:**
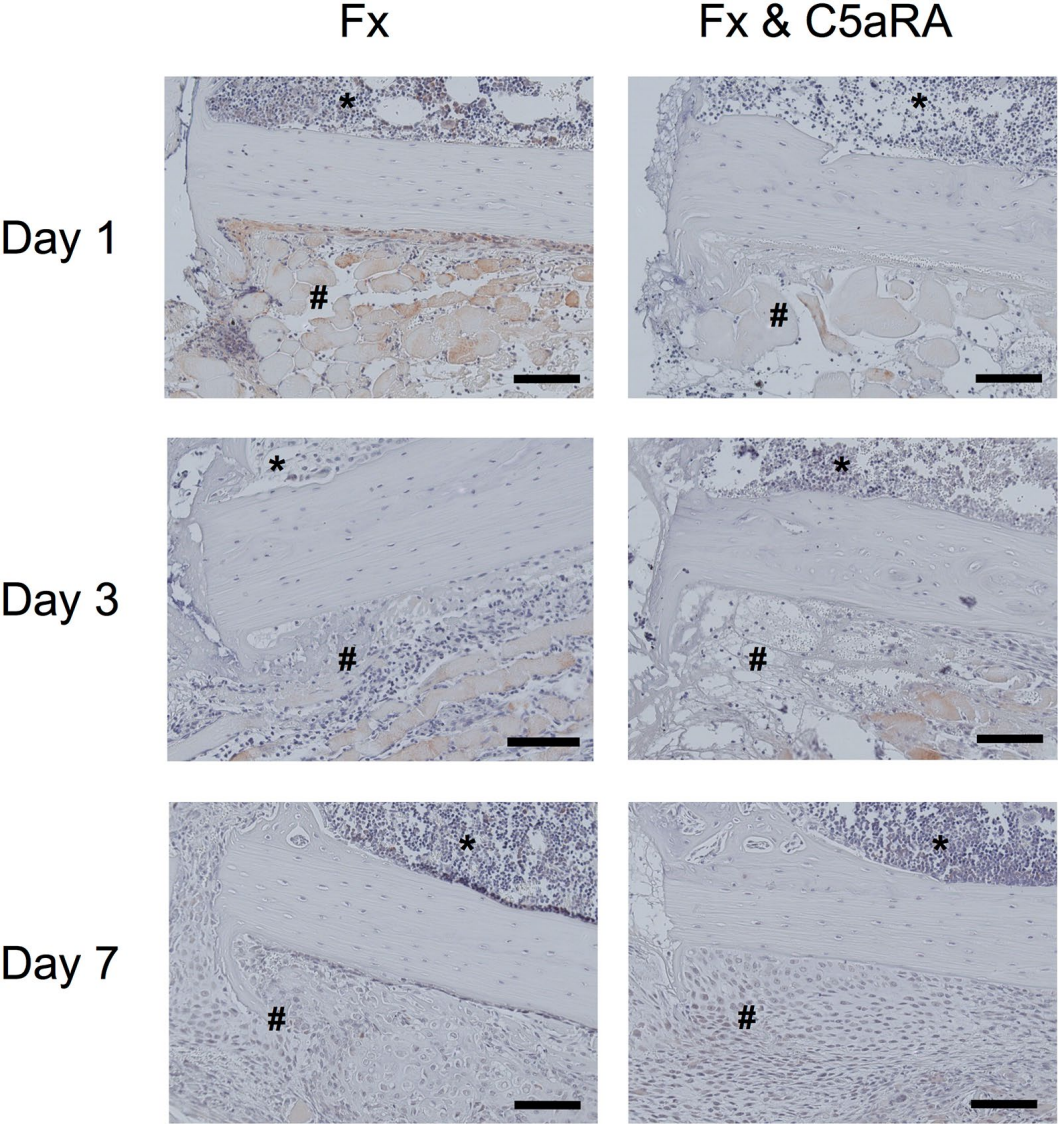
Immunostaining of IL-6 on day 1, 3 and 7 in tissue sections of control animals (Fx) and after administration of C5aRA (Fx & C5aRA) showing the callus region in detail (fracture gap on the* left*, cortex in the* middle*, bone marrow on* top* (*) and periosteal callus (*#*) on the* bottom* of the image. Size of the *scale bar* = 100 µm (200-fold magnification)

Additional examination of IL-6 distribution in the callus area displayed a strong staining of control animals after 1 day, which was not seen in the group treated with the C5aR-antagonist (Fig. 4). After 3–7 days almost similar IL-6 coloring for both experimental groups was demonstrated.

### Serum C5a measurement

**Fig. 5:**
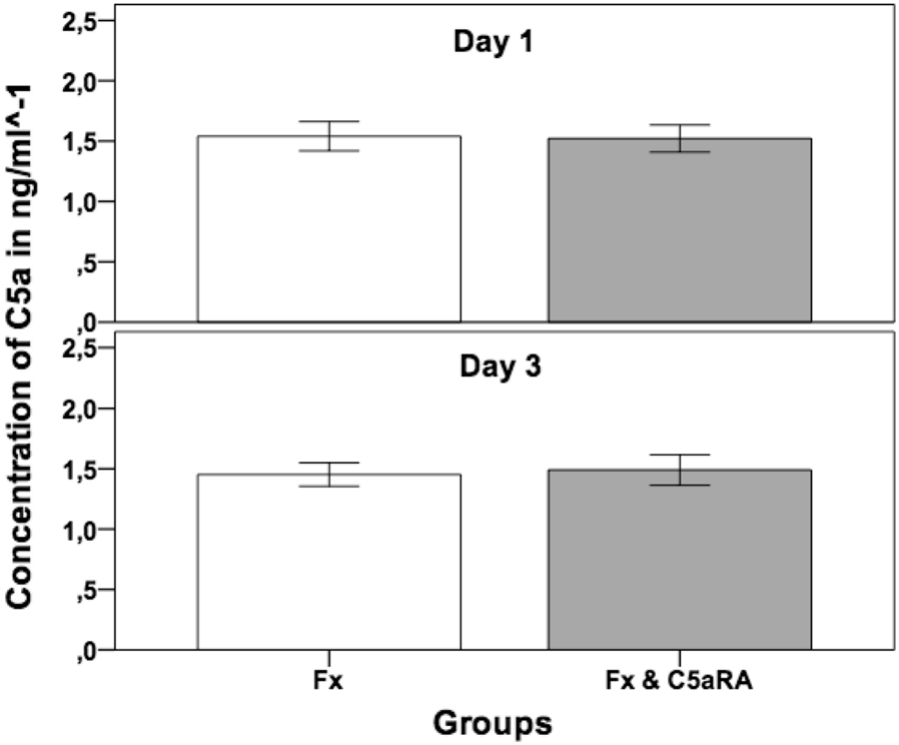
C5a concentration in the serum 1 and 3 days after the osteotomy for both experimental groups (Fx = fracture; Fx & C5aRA = fracture with administration of C5aRA). Results are displayed as mean ± 2 × SEM

The analysis of serum C5a was performed on the early healing stages after 1 and 3 days after osteotomy. C5aRA treatment did not result in a systemic alteration of the amount of generated C5a. In detail, mean value in the fracture group on day 1 was 0.14 ng/ml^−1^ compared to 0.15 ng/ml^−1^ in the group of C5aR-antagonist treatment (Fx & C5aRA). Three days after osteotomy, the amount of C5a was 0.15 ng/ml^−1^ compared to 0.12 ng/ml^−1^ in the group of Fx & C5aRA (Fig. 5).

## Discussion

Recent studies were able to provide first evidence for an involvement of complement in bone biology [19–21].

Our own previous data further suggested that complement may also play a role in fracture healing [10, 22]. We demonstrated that the key complement receptor C5aR was abundantly expressed in the fracture callus of rats not only by immune cells during the early inflammatory phase but also by osteoblasts, chondroblasts and osteoclasts throughout the entire healing period [6].

Besides the increasing evidence for involvement of the complement cascade in bone biology, its exact role (positive/negative) on fracture healing remained unclear.

Various studies were able to show negative side effects of systemic complement activation in diseases like sepsis or blunt chest trauma [14, 15]. But also for musculoskeletal trauma like a femoral osteotomy, negative effects of the complement system with impaired fracture healing after combination with a blunt chest trauma were seen [10]. Systemic administration of a C5a-receptor antagonist abrogated these negative effects, suggesting a detrimental role for the complement cascade in the early and systemic inflammation phase [11]. The reason whether the negative effects on fracture healing were due to an unleashed systemic overexpression of the complement system immediately after trauma or because of its underlying cellular response mechanisms under normal activation remained rather speculative.

To clarify this, in our most recent study we evaluated complement C3- and C5-deficient animals during the 21-day healing period after femoral osteotomy and subsequent external fixation. In this fracture healing model, we were able to demonstrate a beneficial role of the complement system with impaired fracture healing for both complement-deficient strains in the early healing phases and a significantly reduced healing for C5-deficient animals after 21 days. In detail, C5-deficient animals showed a reduced bending stiffness and a smaller callus volume. Furthermore, serum analyses demonstrated activation of C5a in C3−/− mice, suggesting cleavage via extrinsic pathways. Therefore, a crucial role for activation of the terminal complement cascade in successful fracture healing was suspected [5]. In the end, it remained unclear if an early complement blockade mediates the same beneficial effects upon normal fracture healing as it was seen in multiple trauma model with systemic inflammation (blunt chest trauma with femoral osteotomy) [11], especially as some studies stated the possibility of a systemic response after a single femoral fracture [23] even though we were unable to detect a systemic increase of complement activation after an isolated femoral osteotomy in mice [5].

Overall, C5aRA treatment and, therefore, blockade of the early complement response immediately after trauma did not result in a significant alteration of fracture healing as it was seen in the afore-mentioned study of a combined trauma with femoral osteotomy and blunt chest trauma [10, 11]. Whereas biomechanical and histological evaluation after 21 days depicted similar values for both groups mirroring the unaltered fracture healing, a closer look to the results revealed slight differences between the treatment groups. C5aR-antagonist treatment clearly diminished the early inflammatory reaction after 1 day as reflected by the reduced IL-6 immunostaining of the fracture callus. In accordance, the number of peripheral mononuclear cells in the callus was diminished by 16% confirming the reduced early inflammatory reaction on a local level. The systemic C5a concentration was not significantly affected after C5aR blockade. This was not surprising, as our previous study showed no systemic increase of C5a activation in the identical experimental setup [5] with the activation of complement strictly limited on a local level at the fracture site.

In reference to the available literature and our own previous studies, the effects of the complement system should be clearly distinguished between its local and systemic effects.

Locally, fracture healing seems to be dependent on a functional complement system as it was shown in own studies with complement-deficient animals [5]. While short abrogation of complement activation immediately after fracture does not alter the local regeneration processes significantly, the blockade of complement exerts positive effects on fracture healing in a scenario of severe trauma which induces systemic inflammation. Several studies were able to show that systemic complement activation may lead to deleterious effects and impaired fracture healing [10, 15]. Here, immunomodulation with a C5aR-antagonist abrogated the negative effects of the complement activation completely [11].

## Conclusion

Whereas former studies demonstrated that complement inhibition with a C5aR antagonist could abolish the negative effects of posttraumatic inflammation on fracture healing after severe trauma, the present study showed that the same treatment does not influence fracture healing in the absence of a severe injury. These findings implicate that the therapeutic success of complement inhibition on fracture healing depends on the activation state of the complement system after injury.

## Abbreviations

ABSMR: American Society for Bone and Mineral Research
BV: bone volume
BV/TV: bone volume/tissue volume
C3: complement factor 3
C3-/-: complement factor 3 knock-out
C3aR: complement factor 3a receptor
C5: complement factor 5
C5a: complement factor 5a
C5aR: complement factor 5a receptor
C5aRA: complement factor 5a receptor antagonist
CT: computer tomography
μCT: micro-computed tomography
EDTA: ethylene diamine tetraacetic acid
ELISA: enzyme-linked immunosorbent assay
IgG: immunoglobulin G
IL-6: interleukin 6
PMN: polymorphonuclear leukocytes
RA: rheumatoid arthritis
ROI: region of interest
SLE: systemic lupus erythematosus
SEM: standard error of the mean
TV: tissue volume
